# Neurorehabilitation Insights in 2024: Where Neuroscience Meets Next-Gen Tech

**DOI:** 10.3390/brainsci15101043

**Published:** 2025-09-25

**Authors:** Giovanni Morone, Rocco Salvatore Calabrò

**Affiliations:** 1 Department of Life, Health and Environmental Sciences, University of L’Aquila, 67100 L’Aquila, Italy; 2 Santa Lucia Foundation, Scientific Institute for Research, Hospitalization and Health Care (IRCCS), 00179 Rome, Italy; 3 Neurorehabilitation Unit, IRCCS Neurolesi Center “Bonino-Pulejo” 98123 Messina, Italy

Neurological disorders are among the leading causes of disability in industrialized countries. While rehabilitation can help some patients regain autonomy in daily activities, full recovery remains limited to a small proportion [1]. This challenge continues to drive the search for more effective therapeutic approaches, fueled by both clinicians and stakeholders. The push for innovation extends beyond the content of neuromotor therapies to include the methods, settings, and overall organization of neurological rehabilitation services—both in developed and developing countries. The importance of rehabilitation is strongly emphasized by the World Health Organization (WHO), which positions rehabilitation as a central health strategy for the 21st century through its Rehabilitation 2030 initiative. This global agenda recognizes rehabilitation as essential for achieving universal health coverage and optimizing how populations function across the human lifespan [2].

At the forefront of this transformation are emerging technologies, which play a pivotal role in advancing our understanding and delivery of neuromotor therapies—though not without challenges. First, technologies such as advanced functional imaging and neurophysiological tools have deepened our insights into neuroplasticity and recovery mechanisms across various neurological conditions. Second, innovations like robotics, virtual reality, brain–computer interfaces, and neuromodulation are enhancing the effectiveness and personalization of neuromotor interventions. Third, digital health solutions, including telerehabilitation, are expanding access to therapy beyond hospital settings, reaching patients in community and home environments [3].

However, several barriers remain. A longstanding debate persists around the role of technology in care—whether it is the tool or the therapeutic principles behind it that drive outcomes. Additionally, usability issues and the often slow translational pipeline from research to clinical practice hinder the timely adoption of cutting-edge technologies. These challenges underscore the need for a human-centered, integrated approach to rehabilitation that balances innovation with accessibility and clinical relevance.

The aim of this special collection is to provide an update on the main techniques and training protocols currently used in the multidisciplinary neurorehabilitation approach with regard to innovation technologies and innovative methods/techniques.

For these reasons, an increasing number of research studies and randomized clinical trials are pursuing new technologies and methods/techniques to improve rehabilitation efficacy with the aim of adapting current guidelines to consider rehabilitating individuals suffering from a disability of neurological origin.

In this Special Issue, a systematic review investigated the efficacy of immersive virtual reality in children affected by cerebral palsy (Contribution 1). This review highlights that immersive VR is a safe treatment modality even in younger subjects, suggesting that it is time to separately treat evidence from non-immersive and immersive virtual reality. The other systematic review investigated the effectiveness of using mirror therapy in patients with peripheral paralysis of the seventh cranial nerve. To this aim, it is important to understand the potential efficacy of neurocognitive techniques even in peripheral neurological pathologies like Bell’s palsy (Contribution 2). In the near future, the results of a systematic review will shed light on the effectiveness of speed-based interventions in reducing bradykinesia among individuals with Parkinson’s disease. The authors of this review have already published a detailed protocol outlining their methodology and objectives (Contribution 3)

A distributed form of constraint-induced movement therapy (CIMT), involving fewer hours per day over an extended period, has been shown to be both feasible and effective for individuals with chronic stroke in outpatient rehabilitation settings. Treatment protocols demonstrated improvements in upper limb use, motor function, mood, and quality of life (Contribution 4).

The feasibility of immersive virtual reality (VR) in combination with robot-assisted gait training (RAGT) was investigated in individuals with neurological conditions, including cerebrovascular accidents and spinal cord injury. The results showed that 3D immersive and more ecologically valid feedback during RAGT enhanced participants’ motivation and adherence to training—particularly when the VR application was enjoyable and not cognitively demanding (Contribution 5). The effectiveness of two models of telerehabilitation, with a robot and without a robot, was explored in arm recovery in individuals with subacute stroke, highlighting the potential of telerehabilitation interventions (Contribution 6). Robots in rehabilitation are not only useful for improving training effectiveness but also for improving prognosis accuracy when combined with a machine learning algorithm that supports recovery prediction (Contribution 7). Widuch-Spodyniuk’s article highlights the need for psychological evaluation in patients with spinal cord injury by finding a correlation between the presence of neuroticism and spasticity. The authors emphasize that the type of therapy performed (conventional or robotic) does not alter the relationship between neuroticism and spasticity (Contribution 8).

The purpose of the study by Maggio et al. was to evaluate the impact of VR-based training on emotional self-efficacy (associated with cognitive dysfunction) in multiple sclerosis patients and to explore potential gender differences in these outcomes. Authors found that gender may influence the effectiveness of VR-based rehabilitation, which could inform more tailored approaches for emotional and cognitive rehabilitation in MS (Contribution 9).

Zaldumbide-Alcocer and colleagues demonstrated that LEGO^®^-based therapy (LEGO^®^ B-T), a neurohabilitation intervention targeting cognitive impairment, is effective in pediatric populations with epilepsy and associated cognitive dysfunction (Contribution 10).

The articles featured in this Special Issue offer a compelling snapshot of how innovative technologies are transforming the field of neurorehabilitation. They highlight cutting-edge advancements that are enhancing therapeutic outcomes for individuals affected by neurological disorders such as stroke, spinal cord injury, and traumatic brain injury. From robotics and virtual reality to brain–computer interfaces and telerehabilitation, these technologies not only improve motor and cognitive recovery but also increase patient engagement, accessibility, and care personalization. Collectively, the contributions underscore the growing potential of technology-driven interventions to reshape the future of neurological rehabilitation.

## References

[1] Huang Y., Li Y., Pan H., Han L. (2023). Global, regional, and national burden of neurological disorders in 204 countries and territories worldwide. J. Glob. Health.

[2] Vaughn S., Cournan M. (2024). World Health Organization Rehabilitation 2030: Call to Action Update. Rehabil. Nurs..

[3] Bonanno M., Calabrò R.S. (2023). Bridging the Gap between Basic Research and Clinical Practice: The Growing Role of Translational Neurorehabilitation. Medicines.

